# Supramaximal Isometric Holds Enhance Squat Performance: An Integrated Analysis of Barbell Kinematics, Force Production, and Postural Stability

**DOI:** 10.3390/sports14090413

**Published:** 2026-09-18

**Authors:** Álvaro Ocaña-García, Matías Gómez-García, José Manuel Herrero-Atiénzar, José María López-Gullón, Bárbara Bonacasa, Adrián Bayonas-Ruiz

**Affiliations:** 1 Department of Physical Activity and Sport, Faculty of Sports Sciences, Campus de Excelencia Internacional de Ámbito Regional (CEIR) Campus Mare Nostrum (CMN), University of Murcia, 30720 Santiago de la Ribera, Spain; alvaro.ocanag@um.es (Á.O.-G.); luchamurcia@um.es (J.M.L.-G.); 2 UMUSport Research Group, Faculty of Sports Sciences, Campus de Excelencia Internacional de Ámbito Regional (CEIR) Campus Mare Nostrum (CMN), University of Murcia, 30720 Santiago de la Ribera, Spain; 3 Weightlifting Federation of Murcia, 30158 Murcia, Spain; manuher7@gmail.com; 4 Spanish Karate National Team, 28043 Madrid, Spain; matiasgomezgarcia22@gmail.com; 5 High Performance Centre Infanta Cristina, Spanish Olympic Committee, Los Narejos, 30710 Murcia, Spain; 6 Department of Physiology, Faculty of Medicine, Edificio LAIB/Departamental, Campus de Excelencia Internacional de Ámbito Regional (CEIR) Campus Mare Nostrum (CMN), Campus CC Salud-El Palmar, 30120 Murcia, Spain; bonacasa@um.es; 7 Research Group of Physical Exercise and Human Performance, Edificio LAIB/Departamental, Campus de Excelencia Internacional de Ámbito Regional (CEIR) Campus Mare Nostrum (CMN), Campus CC Salud-El Palmar, 30120 Murcia, Spain

**Keywords:** post-activation performance enhancement, isometric pre-conditioning, force plate, barbell velocity, center-of-pressures, powerlifting

## Abstract

Optimizing acute performance during strength training and competition has become an important objective for coaches and athletes. This study aimed to analyze the acute effect of supramaximal isometric holds (SMHs) on squat performance integrating barbell kinematics, force production, and postural stability. Fourteen resistance-trained subjects completed six experimental sessions encompassing squats at 60% and 80% of one-repetition maximum (1RM) following a 10 s SMH at 110% of 1RM with either 1 min or 5 min recovery, or in a control condition (CON) with no SMH. Barbell kinematics were assessed using a linear position transducer, as were vertical ground reaction force, inter-limb asymmetries, and center-of-pressure metrics via dual-force platforms. An SMH with 1 min recovery produced significant increases in mean propulsive velocity at both intensities compared to CON (+0.05 m·s^−1^; *d* ≥ 0.94), a higher rate of force development at 80% of 1RM (*p* < 0.01), and non-significantly increased peak concentric force at 80% of 1RM (*p* = 0.056). An SMH with 5 min recovery had little to no effect. No significant differences were found in inter-limb force asymmetries or center-of-pressure metrics. A 10 s SMH coupled with a 1 min recovery period is a practical and costless strategy to enhance squat performance in strength-trained individuals through increased barbell velocity, force production, and unchanged postural stability.

## 1. Introduction

Optimizing acute performance during strength training and competition has become an important objective for coaches and athletes. Priming strategies are increasingly used to optimize acute performance before strength and power tasks. However, the integrated mechanical response to supramaximal isometric priming remains poorly characterized.

Supramaximal isometric holds (SMHs) are a priming strategy for athletes aiming to improve performance in the squat exercise [1]. The protocol involves unracking a barbell with supramaximal load (e.g., >100% of one-repetition maximum) and maintaining the squat starting position for a prescribed duration before reracking the load [1,2]. Supramaximal isometric holds belong to the broader framework of post-activation performance enhancement (PAPE) strategies, which involve high-intensity conditioning activities [3,4]. Dynamic PAPE protocols typically involve a low number of repetitions with heavy loads before an explosive task, whereas isometric approaches rely on sustained high-intensity contractions [5,6]. Although dynamic PAPE strategies have been extensively investigated, the effects of isometric pre-conditioning remain comparatively underexplored [7]. Nevertheless, both approaches have demonstrated the potential to acutely enhance subsequent performance [4,8].

The mechanisms proposed to underlie PAPE include increased neural drive, enhanced muscle activation, and improvements in contractile responsiveness [8,9,10]. However, the magnitude of the response appears highly dependent on the balance between potentiating and fatiguing stimuli [3,9,11]. Consequently, effective PAPE programming requires careful manipulation of conditioning intensity, volume, and recovery duration [3,9]. Within isometric conditioning protocols, evidence suggests that potentiation may depend more strongly on contraction intensity than duration [12]. In line with this, Eon et al. reported a significant relationship between conditioning intensity and post-activation potentiation amplitude [12]. More recently, supramaximal walkouts (SMWs) performed at 110% of one-repetition maximum (1RM) attenuated the decline in subsequent squat barbell velocity at 92.5% 1RM compared with a control walkout at 30% 1RM [1].

Barbell velocity assessment enables real-time monitoring of neuromuscular performance during resistance exercise. In particular, changes in mean propulsive velocity (MPV) have shown high sensitivity to acute performance fluctuations and chronic training adaptations [13,14,15]. In addition, eccentric kinematics [16,17] and force-platform-derived metrics [18] further characterize movement mechanics, force production, and postural stability during squat execution.

To date, research examining PAPE predominantly focuses on athletic tasks such as jumping, sprinting, and change-of-direction performance [7], whereas its effects on strength-specific performance and exercise mechanics remain insufficiently understood [1,6,7,19,20]. Prior studies evaluating squat-based PAPE protocols have mainly assessed performance in sets to failure [19,20]. Among them, Rodrigues et al. [20] observed similar mean and peak concentric velocities following heavy squat conditioning compared with a general warm-up condition. More recently, Griest et al. [1] demonstrated an attenuated decline of barbell velocity after SMW. However, SMWs include additional locomotor demands during the walkout and reracking phases, which may increase fatigue and alter the balance between potentiating and fatiguing stimuli compared with stationary supramaximal holds [1,3,9]. Moreover, no previous investigation has incorporated force-platform-derived metrics to examine the effects of supramaximal isometric pre-conditioning on force production characteristics and postural stability during the squat exercise.

Despite their widespread implementation, the effects of SMH on subsequent squat performance remain poorly characterized. Therefore, the aim of this study was to examine the effects of SMH on subsequent barbell kinematics, force production, and postural stability in the back squat exercise in resistance-trained individuals.

## 2. Materials and Methods

### 2.1. Study Design

**Figure 1:**
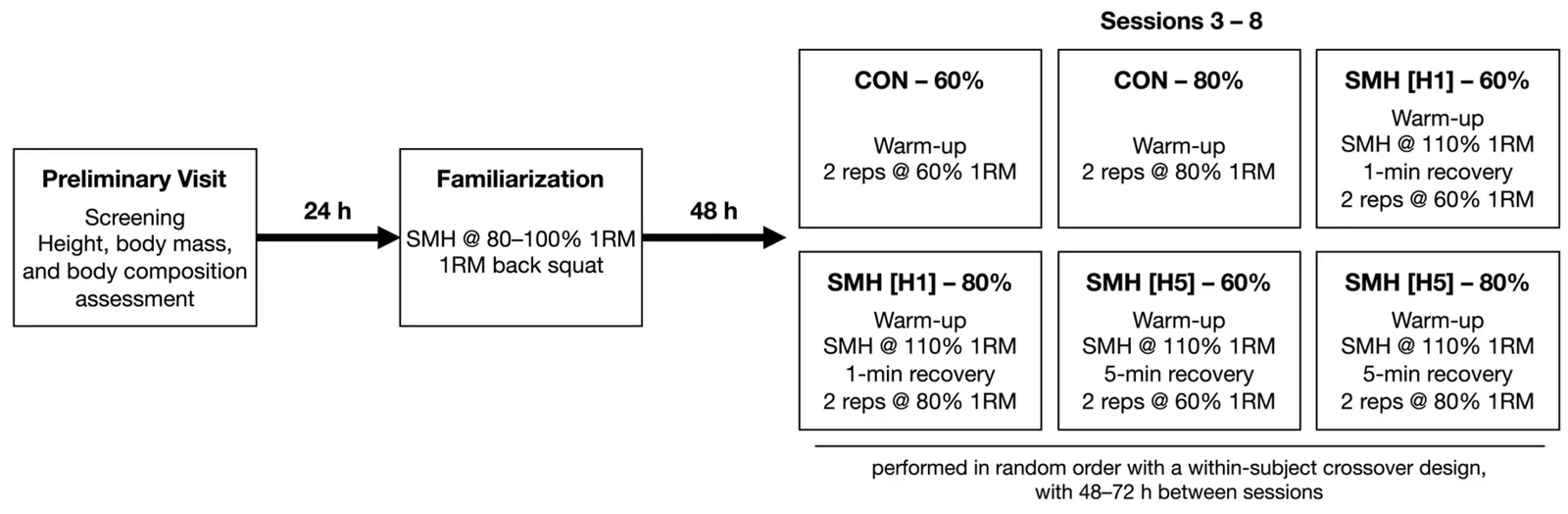
Experimental design. CON: control condition with no SMH; H1: SMH coupled with a 1-min recovery interval; H5: SMH coupled with a 5-min recovery interval SMH: supramaximal hold; 1RM: one-repetition maximum; @: “at” a given percentage of 1RM.

A randomized, within-subject crossover design (Figure 1) was utilized to examine the acute effects of SMHs on back squat performance, barbell kinematics, and postural stability. During the first visit, participants completed screening procedures, provided informed consent, and underwent height and body mass assessments. The second visit served as a familiarization session and included 1RM testing.

Visits 3–8 corresponded to the experimental trials. After a standardized warm-up, subjects performed squats against 60% or 80% of 1RM after a 10 s SMH at 110% of 1RM followed by either a 1 min (H1) or 5 min (H5) recovery, or in a control condition (CON) with no previous SMH. The loads analyzed were selected on the basis of representing moderate and high relative intensities commonly used in resistance-training practice [21], with which power output is maximized in the squat [22]. The warm-up consisted of 5 min of jogging at an easy self-selected pace, 5 min of joint mobilization, and four squat sets at 30%, 40%, 50%, and 60% of 1RM (of six, four, two, and one repetition, respectively). Two repetitions were performed in each experimental condition, and the best repetition according to the fastest MPV criteria was analyzed [23]. Simple randomization was used to determine session order, ensuring unbiased distribution of testing sequences across participants. Sequences were generated with no restrictions in Microsoft Excel and randomly assigned to a participant identifier code. Participants were unaware of the testing procedure corresponding to each day until finishing the warm-up to avoid potential anticipation effects [1]. Assessments were conducted at the same time of day and in the same gym facilities. Experimental sessions were separated by 48–72 h and completed within a 15-day period. Adverse events (muscle discomfort or injuries) were recorded non-systematically according to participants self-reporting. The study was conducted in accordance with the Declaration of Helsinki and approved by the Bioethics Committee of University of Murcia (approval number: 2325/2019).

### 2.2. Participants

**Figure 2:**
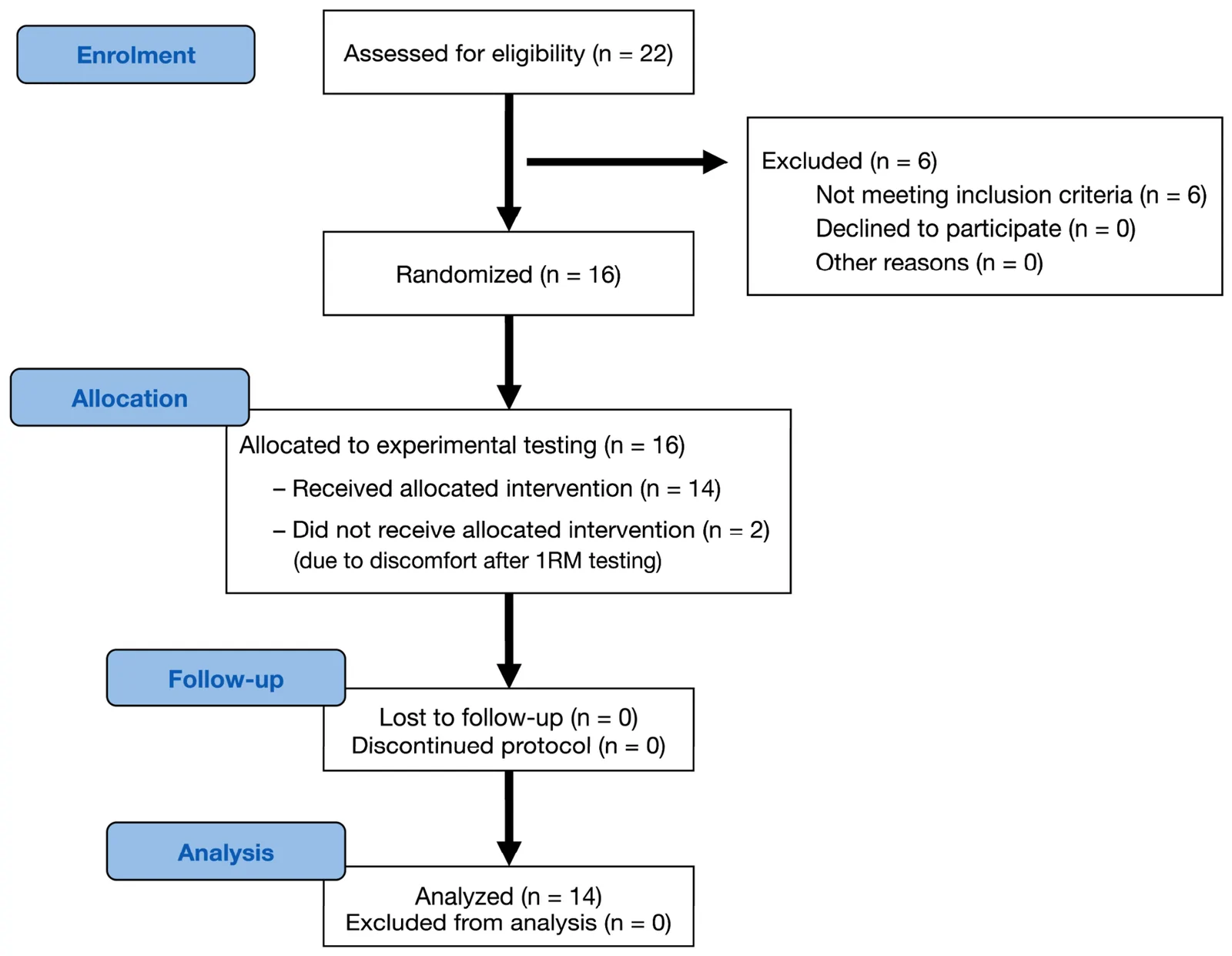
CONSORT flow diagram.

Fourteen healthy strength-trained participants (11 men, 3 women; age range: 18–24 years; body mass 69 ± 10 kg; height 171 ± 7 cm; resistance training experience: 3 ± 1 years; squat training experience: 2–5 years) with 1RM strength of 100 ± 17 kg and a relative strength ratio (1RM normalized to body mass) of 1.50 ± 0.18 (range 1.22–1.77) volunteered to take part in this study (Figure 2). Inclusion criteria were (i) absence of neuromuscular and musculoskeletal disorders; (ii) a minimum of two years of training experience in the squat exercise; (iii) a minimum of 1 squat session per week for at least one year prior to the study.

Subjects were instructed to maintain their typical dietary and sleep habits and to refrain from consuming stimulants and alcoholic beverages [10]. In addition, they were instructed to maintain their usual sport-specific training and to refrain from additional resistance exercises during the study course and 48 h prior to 1RM testing. All were international level wrestlers and karatekas [24].

An a priori sample size estimation was performed for repeated-measures ANOVA within factors using G*Power 3.1 (Heinrich-Heine-Universität, Düsseldorf, Germany). A moderate-to-large effect size (f = 0.30), α = 0.05, and power = 0.80 were utilized on the basis of previous findings [1]. Assuming a repeated-measures correlation of 0.70 and a nonsphericity correction ε = 1, the required sample size was 13 participants. No interim analyses were planned or performed, and no stopping guidelines were prespecified.

### 2.3. Procedures

#### 2.3.1. Individual 1RM Strength Determination

The individual load–velocity relationship and 1RM strength were determined by means of a progressive loading test described elsewhere [25]. Subjects lifted the bar with maximal intended concentric velocity [26,27]. For each absolute load, only the fastest repetition according to MPV was considered for subsequent individual load–velocity profile analysis [27]. The individually determined velocity for each %1RM was used to precisely adjust the load in each experimental session [25,28]; before each test, warm-up loads, which were common to all testing sessions, were analyzed and adjusted as necessary to ensure that the MPV aligned with the expected value derived from each subject’s load–velocity profile (±0.02 m·s^−1^) [29] and to finally select the appropriate load for each session based on estimated daily 1RM [28].

#### 2.3.2. Supramaximal Isometric Holds and Squat Technique

**Figure 3:**
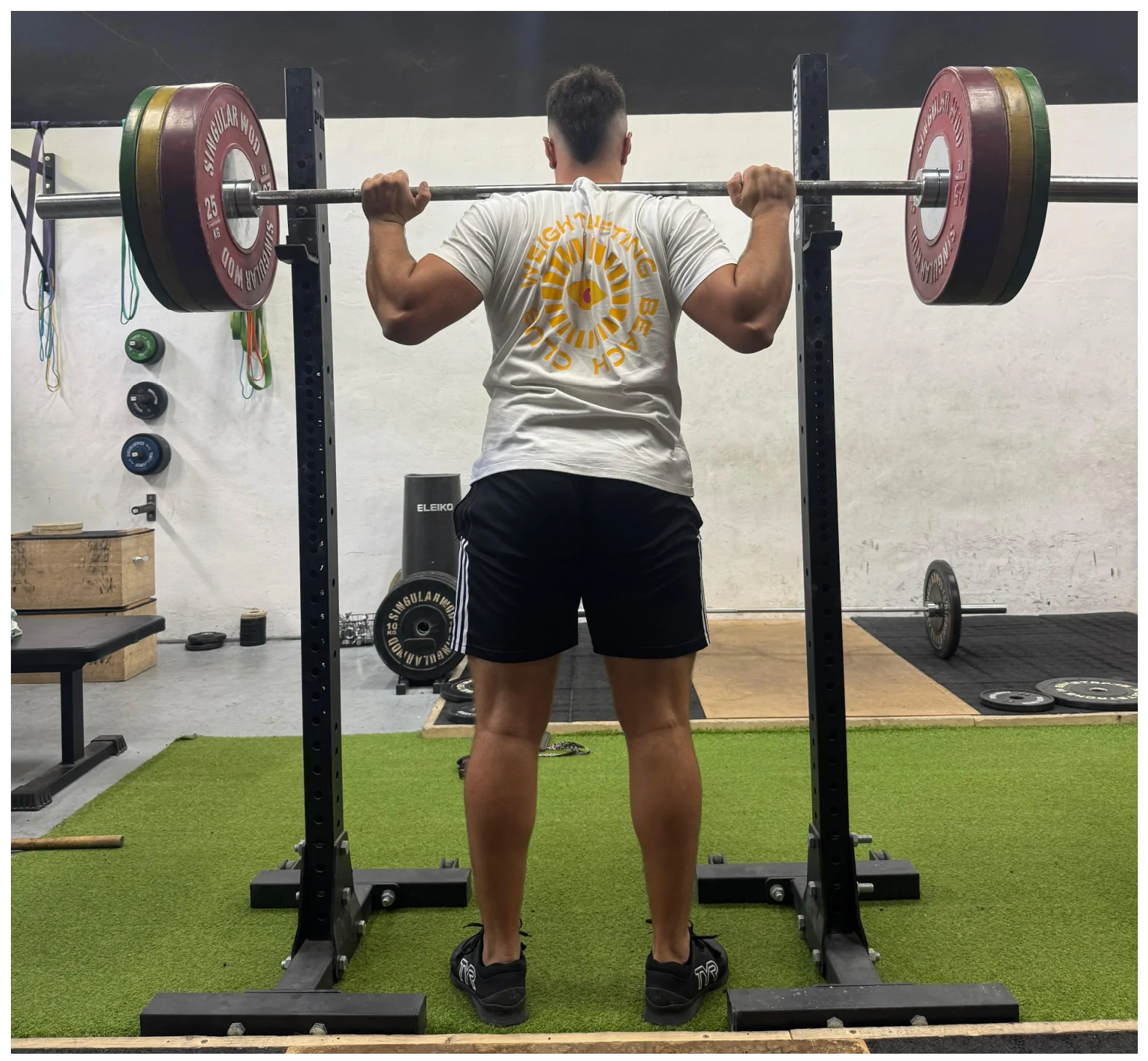
Illustration of the set-up utilized for supramaximal isometric holds and testing.

Subjects completed a 10 s SMH before the corresponding experimental conditions, which involved unracking a barbell loaded to 110% of 1RM and maintaining an upright posture corresponding to the initial position of the squat exercise (Figure 3) [1]. The same weight based on 110% of the baseline 1RM determination was used throughout the study. Rack height was individually adjusted to minimize the range of motion and effort required during bar unracking. Following unracking, participants were required to keep the upright posture without taking any additional steps (without a walkout). After 10 s, participants were prompted to rerack the bar and initiated the corresponding passive recovery period, which was performed seated or standing as preferred. Two investigators loaded the plates in the barbell during all testing sessions, including unloading from 110% of 1RM after the SMH. A familiarization session featuring identical warm-up, unracking, 10 s hold, and reracking of three increasing loads corresponding to their recent (<6 months) 80–110% 1RM were performed prior to testing. The intensity of the SMH was selected on the basis of the previous literature supporting the notion that isometric preconditioning is maximized when contractions reach 80–100% of MVIC [12,30] and matching the method used in a similar study [1]. Duration was selected on the basis of a recent meta-analysis which suggested that the potentiation effect of isometric strategies peaks at 10 s [8].

#### 2.3.3. Measuring Equipment and Variables Analyzed

A power rack (SingularWOD; Madrid, Spain) and Olympic bar (length 220 cm, grip diameter 28 mm, weight 20 kg) (Eleiko, Sport AB; Halmstad, Sweden) were used in all sessions. Extra load was added by sliding on calibrated weight plates (Eleiko, Sport AB). Repetitions were recorded using a linear velocity transducer with a sampling frequency of 1000 Hz (T-Force System, Ergotech, Murcia, Spain). This technology has been validated for measuring barbell velocity in the back squat [31,32], demonstrating outstanding reproducibility and reliability [33]. Data from MPV, mean velocity (MV), eccentric peak velocity (PV), and mean and peak eccentric power (MP and PP) were obtained.

Vertical ground reaction force and center-of-pressure (COP) positioning data were collected using a dual-force plate system at a sampling frequency of 1000 Hz (Hawkin Dynamics; Westbrook, ME, USA) [34]. Peak force (PF: highest instantaneous force registered at a particular instant –1 ms–); mean force (MF: average force registered); inter-limb asymmetries in MF (inter-limb difference relative to the highest MF registered by one leg); and peak rate of force development (RFD) with a 50 ms moving window were obtained.

Stability was assessed by means of COP variability in the medio-lateral (ML) and antero-posterior (AP) planes [35,36] by calculating COP sway (SD of data) [37] from the raw (unfiltered) COP positioning files extracted from the Hawkin Dynamics System [34]. The variability in COP is reported relative to foot size [36] (e.g., a 2 cm COP sway in the AP plane corresponded to a 10% variation for a subject with a 20 cm long foot). All variables were obtained for the eccentric and concentric phases of every repetition.

### 2.4. Force–Time Data Processing and Phase Segmentation

Eccentric and concentric phases within the force-platform recordings were identified post hoc by combining the raw force–time data with the phase durations obtained from the linear position transducer. All repetitions were processed by the same investigator (AOG), blinded to the condition processed, following a standardized procedure.

Raw force–time curves were visually inspected using the Hawkin Dynamics web interface. The segmentation of the eccentric phase was performed manually using (i) the duration extracted from the linear position transducer; (ii) the first time point at which force showed a sustained decrease from the quiet-standing baseline toward the minimum force attained during the descent [38,39]; and (iii) the return point (i.e., the point of eccentric–concentric coupling identifiable as the instant with the highest recorded vertical ground reaction force followed by an acute decrease [39]). The investigator selected the start point as proposed [38], verified that the end of the time frame corresponded to the eccentric phase, as extracted from the linear position transducer coinciding with the return point, and corrected the start point where necessary. The concentric-phase duration determined by the linear position transducer was then applied sequentially from the return point, delimiting the phase end. Force-derived variables and COP metrics were subsequently separately calculated for each phase.

### 2.5. Statistical Analyses

Normality of the data was assessed through inspection of Q–Q plots and Shapiro–Wilk tests. Standard statistical methods were used for the calculation of means, standard deviations (SDs), and 95% confidence intervals (CIs). A repeated-measures analysis of variance (ANOVA) was conducted to assess differences across the three within-subject conditions (CON, H1, and H5) for the variables of interest separately for each load. The assumption of sphericity was assessed using Mauchly’s test. When the assumption was violated, Greenhouse–Geisser corrections were applied. When significant main effects were found, pairwise comparisons were performed using Bonferroni-adjusted tests. Significance was accepted at *p* < 0.05. Effect sizes (ESs) were calculated and interpreted using eta partial squared (η^2^_p_) and Cohen’s *d* for paired samples (*d*z). Paired *t*-tests or Wilcoxon signed-rank tests were utilized as appropriate to assess the differences between eccentric and concentric inter-limb asymmetries in force production, and in the eccentric velocities in CON at 60% and 80% of 1RM. Effect sizes were calculated using Cohen’s *d*. The data analyst (ABR) was aware of testing allocation. To minimize the risk of analytical bias, all statistical procedures and outcomes were defined a priori and implemented according to the prespecified analysis plan. Data from two participants who dropped out of the study after 1RM assessment due to muscle discomfort (Figure 2) were eliminated from height, body mass, 1RM, and relative strength analyses. Calculations were performed using a Microsoft Excel spreadsheet and the SPSS software (version 28.0, IBM Corp., Armonk, NY, USA).

## 3. Results

### 3.1. Barbell Kinematics

**Figure 4:**
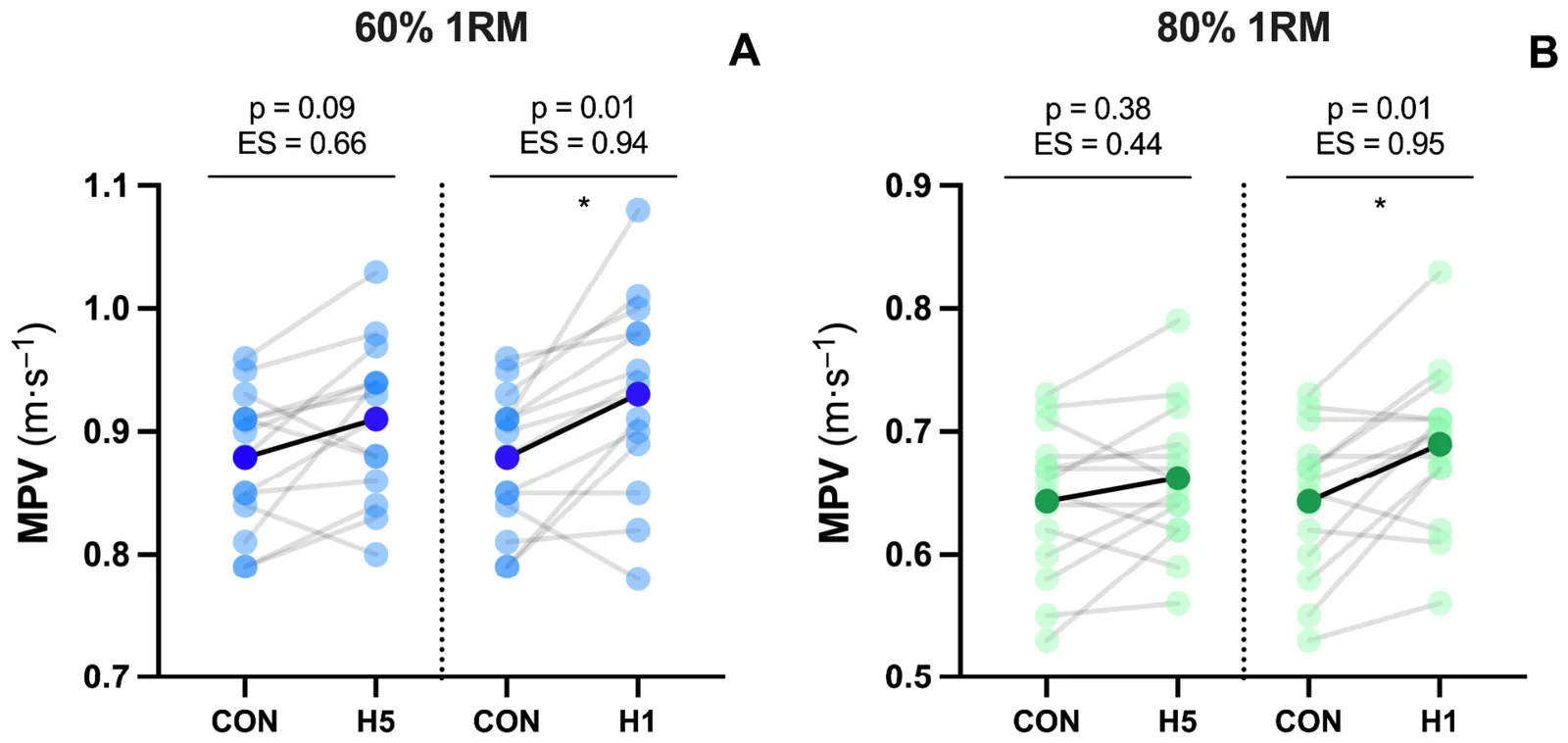
Mean propulsive velocity (MPV) during the squat exercise at 60% (**A**) and 80% (**B**) of 1RM under control conditions (CON) and following a supramaximal isometric hold with 5 min (H5) or 1 min (H1) of recovery. Light-colored circles and connecting lines represent individual comparisons between conditions, whereas dark-colored circles and black lines indicate group mean responses. Statistical significance and paired effect sizes (ES, Cohen’s *d*) are displayed above each comparison. * Significant (*p* < 0.05) paired differences.

Bar velocity increased following SMH. Significant effects of condition were observed for both MV and MPV at 60% (*p* < 0.01, η^2^_p_ = 0.29 and 0.32, respectively) and 80% of 1RM (*p* < 0.01, η^2^_p_ = 0.30 and 0.36). Post hoc analyses revealed higher MPV after H1 compared with CON at both 60% and 80% of 1RM (+0.05 m·s^−1^; CI: 0.01–0.09 and 0.01–0.08; *d* = 0.94 and 0.95, respectively; Table 1, Figure 4). No significant differences were observed between H5 and CON, although moderate effect sizes were detected at 60% of 1RM (*d* = 0.66).

ROM remained unchanged across conditions (Table 1). At 60% of 1RM, eccentric velocity and power variables did not differ between conditions. At 80% of 1RM, eccentric PV was higher after H1 than in CON (+0.09 m·s^−1^, CI: 0.01–0.17, dz = 0.77), whereas eccentric MV, MP, and PP showed moderate, non-significant increases after H1 (all *p* ≥ 0.06; Table 1).

### 3.2. Force Production

**Figure 5:**
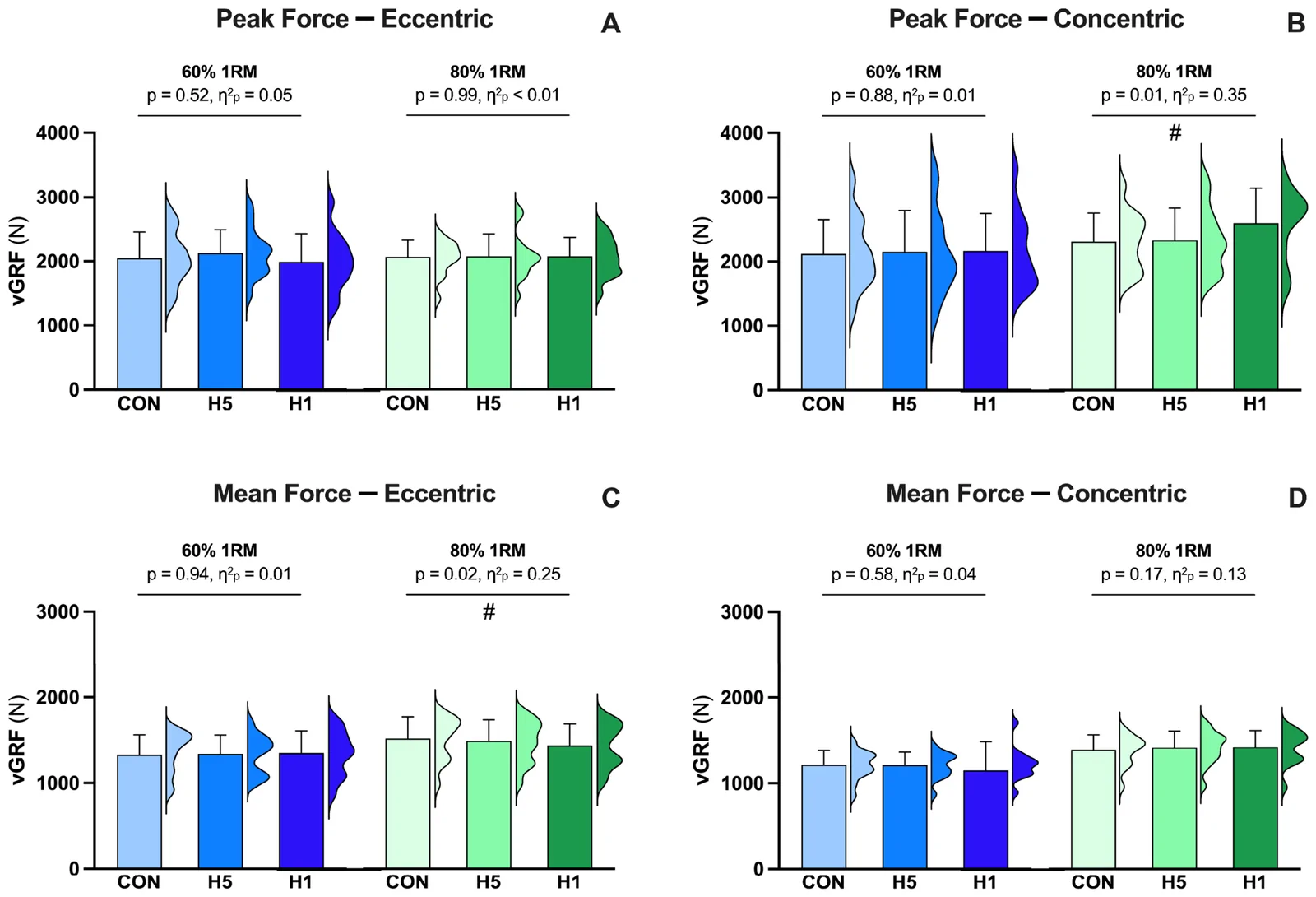
Peak (**A**,**B**) and mean (**C**,**D**) vertical ground reaction force (vGRF) during the eccentric and concentric phases of the squat exercise at 60% and 80% of 1RM under control conditions (CON) and following a supramaximal isometric hold with 5 min (H5) or 1 min (H1) of recovery. Bars represent mean ± SD, whereas half-violin plots show the distribution of individual responses. Statistical significance and effect sizes are displayed for omnibus comparisons. Number signs (#) indicate significant (*p* < 0.05) omnibus differences at the corresponding intensity and phase of the lift. Effect sizes are reported using eta partial squared (η^2^_p_).

Peak eccentric force did not differ between conditions at either load. Mean eccentric force and peak concentric force showed condition effects at 80% of 1RM, with post hoc comparisons indicating non-significant lower mean eccentric force (*p* = 0.060) and higher peak concentric force (*p* = 0.056) after H1 relative to CON (Figure 5). Peak concentric RFD increased at 80% of 1RM (*p* < 0.001, η^2^_p_ = 0.45), with H1 (28,690 ± 10,709 N·s^−1^) producing greater values than CON (20,523 ± 8366 N·s^−1^) with a large effect size (+8167 N·s^−1^, 95% CI: 3171 to 13,164, *d*z = 1.20, *p* < 0.01), whereas H5 (21,150 ± 10,051 N·s^−1^) did not differ from CON. No effects were observed at 60% of 1RM: 21,523 ± 10,051 N·s^−1^ in CON, 22,522 ± 12,187 in H1, and 25,274 ± 10,343 in H5 (*p* = 0.54).

### 3.3. Interlimb Asymmetries

Mean inter-limb asymmetry was greater during the concentric than eccentric phase at both loads (all *p* < 0.05). At 80% of 1RM, concentric asymmetry showed moderate-to-large reductions after SMH (CON: 26%, H5: 22%, H1: 14%), although the overall effect did not reach significance (*p* = 0.10, η^2^_p_ = 0.16). This reduction was driven by an increase in mean force production of the weaker limb following H1 compared with CON (674 ± 120 vs. 604 ± 106 N, respectively, *p* = 0.016), whereas the force produced by the stronger limb remained unchanged. No differences were observed at 60% 1RM.

### 3.4. Postural Stability

**Figure 6:**
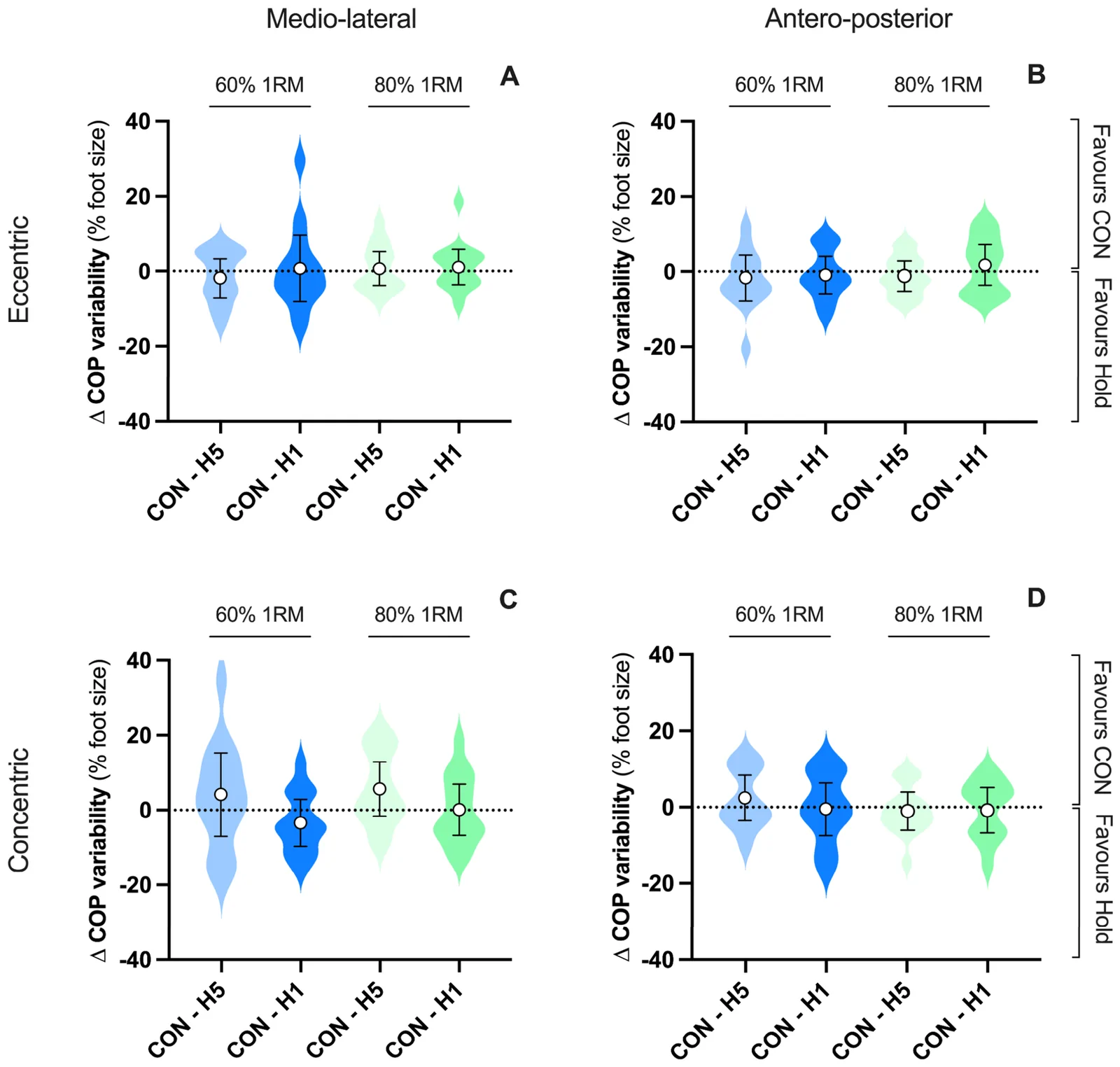
Differences (∆) in center of pressure (COP) variability in the medio-lateral (**A**,**C**) and antero-posterior (**B**,**D**) planes during the eccentric and concentric phases of the squat exercise at 60% and 80% of 1RM under control conditions (CON) and following a supramaximal isometric hold with 5 min (H5) or 1 min (H1) of recovery. Data are shown as violin plots (kernel density estimation) with mean differences and ± 95% confidence intervals.

No significant differences in COP variability were observed across all loads and conditions, in both the eccentric and concentric phases, and both medio-lateral and antero-posterior planes (all *p* > 0.05, Figure 6).

## 4. Discussion

This study examined the effects of SMH on barbell kinematics, force production, and postural stability in the back squat exercise in resistance-trained individuals. The main finding was that a 10 s SMH coupled with a 1 min recovery interval elicited a significant increase in barbell velocity and force production compared to CON, with no changes in inter-limb asymmetries and unchanged postural stability.

Importantly, squat ROM was consistent across conditions, supporting the interpretation that the observed effects reflected genuine performance changes rather than alterations in squat depth [22].

### 4.1. Concentric Barbell Kinematics

These findings contrast with those of Griest et al. [1], who reported a non-significant reduction in mean velocity following supramaximal walkouts at 110% of 1RM with a 5 min rest prior to squats at 92.5% of 1RM. Several methodological differences may explain these discrepant findings. Unlike stationary SMHs, supramaximal walkouts involve additional locomotor and stabilization demands during walkout and reracking, which may increase fatigue and alter the balance between potentiating and fatiguing stimuli. Given that the effectiveness of PAPE strategies appears highly dependent on this balance [3,9,11], the present findings may suggest that stationary SMHs could provide sufficient potentiating stimulus while minimizing unnecessary fatigue associated with SMW tasks. Future research warrants direct comparisons of both strategies.

Athletes’ strength levels are determinants of post-activation potentiation [11] and influence the PAPE response [3,9]: stronger individuals generally demonstrate faster fatigue dissipation and a broader potentiation window. In this regard, subjects in Griest et al. [1] exhibited substantially greater relative squat strength and training experience than those included in the present study. Nevertheless, despite the higher training status of their cohort, SMWs were not associated with performance enhancements, in contrast with the stationary SMH protocol examined here. Although direct comparisons between studies should be interpreted cautiously, these findings further support the possibility that reducing unnecessary locomotor demands in supramaximal isometric pre-conditioning may be advantageous to optimize the potentiation–fatigue balance.

The PAPE effect of isometric pre-conditioning depends primarily on achieving a sufficiently high contraction intensity (~80–100% of maximal voluntary isometric contraction) rather than on time spent under tension alone [12,30]. Nevertheless, when a sufficient contraction intensity is reached, duration may also modulate the potentiation response, with total isometric durations between 8 and 12 s potentially eliciting the greatest performance enhancement [6]. Meta-analytic data further indicate that the potentiating effect may peak at approximately 10 s, whereas shorter (<8 s) or longer (>12 s) durations appear to produce trivial effects, potentially due to insufficient stimulus or excessive fatigue [8].

In line with this, Rappelt et al. [2] reported no improvements in countermovement jump (CMJ) performance following a 3 s SMH performed at 130% of half-squat 1RM, whereas Jarosz et al. [30] similarly observed no changes in CMJ height after 3 × 9 s isometric contractions. Collectively, these findings suggest that excessively short or prolonged isometric conditioning durations may attenuate the net potentiating response associated with supramaximal squat holds. This may also partly explain the findings observed in the study using SMWs [1], as the combined duration of the walkout and hold phases may have resulted in an excessively prolonged isometric contraction. On the other hand, protocols involving brief (~3 s) SMHs may have provided insufficient stimulus to elicit a meaningful PAPE response [2].

### 4.2. Eccentric Barbell Kinematics

Mean eccentric velocity is a relevant kinematic variable in squat execution [23,27] and is usually set between ~0.45–0.65 m·s^−1^ for back squats in VBT research to ensure homogeneous execution [14,22]. However, contexts outside research may vary and different approaches to the eccentric phase may exist. To evaluate the effect of SMH on eccentric bar kinematics we allowed subjects to perform squats at their preferred eccentric execution. A representative finding was that mean eccentric velocity was significantly higher at 60% 1RM compared to 80% in CON (0.73 ± 0.22 and 0.58 ± 0.14, m·s^−1^, respectively, *p* < 0.05, *d* = 0.60), consistent with previous findings [17]. Mean eccentric velocity at 60% of 1RM in CON was therefore faster than 0.45–0.65 m·s^−1^, and interestingly it remained largely unchanged after SMH. On the other hand, peak eccentric velocity at 80% of 1RM augmented after H1, and a moderate-to-large effect size was also observed for mean eccentric velocity despite not reaching significance (*p* = 0.08). Specifically, values increased from 0.58 m·s^−1^ in CON to 0.66 m·s^−1^ in H1. To our knowledge, this is the first study to evidence a change in eccentric execution following pre-conditioning in squats. It may partially link to the increase in peak concentric RFD observed at 80% of 1RM in H1 as a result of augmented elastic energy storage in the stretch-shortening cycle (SSC) [40,41], suggesting that SMH may elicit a more SSC-dependent squatting strategy in H1.

### 4.3. Force Production

Such PAPE responses have been explained through several proposed mechanisms, including enhanced force production [8,9,10]. Although the underlying mechanisms were not directly measured in the present study, the force-platform findings are consistent with such responses and provide evidence of the effect of SMH on concentric force production and RFD. Specifically, a large effect size was observed in the omnibus comparison of peak concentric force at 80% of 1RM (Figure 5B). Interestingly, peak concentric force was non-significantly higher after H1 compared to CON (+290 N, 95% CI: −6 to 587, *p* = 0.056), whereas it was similar after H5 compared to CON (+26 N, 95% CI: −133 to 185, *p* = 1.00). Despite paired differences were non-significant, these observations might be in line with the notion that the potentiating effects of high-intensity isometric pre-conditioning are most pronounced during the early recovery period (~1 min) compared to longer rest durations [8].

In addition to greater force production, the increase in peak concentric RFD observed in H1 relative to CON (~40% of CON value) highlights an enhanced capacity to rapidly generate force. Previous research suggests that the potentiation mechanism may involve improvements in excitation–contraction coupling and increased calcium sensitivity of the contractile apparatus [3,42], and future research is warranted to assess if such mechanisms could have contributed to the faster force-generating response after SMH.

Interestingly, no significant differences in mean force, peak force, or RFD were detected at 60% of 1RM. One possible explanation relates to the load–power relationship of the full squat. Power output is maximized at approximately 50–60% of 1RM, whereas heavier loads are associated with lower power production [22]. Consequently, when performance is already close to the optimal region of the load–power curve, there may be less potential for further enhancement, although this hypothesis should be studied in future research. In contrast, acute improvements in neuromuscular function may become more apparent at higher relative loads, where force-generating demands are greater.

On the other hand, a significant omnibus effect of condition was found for eccentric mean force at 80% of 1RM. Although paired differences were non-significant: −80 N (95% CI: −179 to 18, *p* = 0.13) in CON–H1, and −29 N (95% CI: −98 to 40, *p* = 0.82) in CON–H5, these findings might be related to the non-significant increase in mean eccentric velocity at 80% of 1RM after SMH [43] (Table 1), while the topic warrants further research.

### 4.4. Inter-Limb Asymmetries

Another noteworthy observation was the reduction in inter-limb force asymmetry following SMH. Although certain asymmetry is inherent to human movement [44], larger between-limb differences may limit the overall force-generating capacity of bilateral tasks [44,45]. At 80% of 1RM, asymmetry decreased from 26% in CON to 14% in H1, accompanied by non-significant moderate-to-large ES (*p* = 0.10). Additional analyses indicated that this change was attributable to increased force production in the weaker limb, whereas force production in the stronger limb remained largely unchanged. To our knowledge, this is the first study to report such a response following a pre-conditioning protocol. Although these findings should be interpreted cautiously, they suggest that the acute effects of SMH may extend beyond increases in overall force production and may also influence the distribution of force production between limbs during back squats.

### 4.5. Postural Stability

Changes in COP variability are utilized to analyze postural stability during back squats [35,36]. Reductions in COP variability are generally interpreted as indicative of enhanced postural control and balance performance [35]. Previous research associated greater COP displacements with poorer balance and reduced movement control during squats [46]. Regarding performance, increased stability likely facilitates a more efficient force transmission by minimizing unnecessary oscillations of the center of mass relative to the base of support [37].

Footwear, fatigue, or strength levels impact COP variability in the squat [35]. However, evidence regarding the effect of pre-conditioning is scarce. Moreover, whether changes in COP variability influence neuromuscular performance in back squats remains unexplored. Footwear, strength levels, and fatigue were consistent across testing conditions in the present study and no effect of SMH on COP variability was observed. The findings support that stability was largely unchanged in both ML and AP planes across all experimental conditions (Figure 6). This suggests that in strength-trained, moderately strong lifters, SMH have no significant effect on stability. Moreover, this may suggest that postural stability had little influence on the improvements in neuromuscular performance, although this should be interpreted cautiously.

### 4.6. Limitations and Future Research

This study is not exempt from limitations. First, although the present findings suggest that stationary SMH may provide a favorable balance between potentiation and fatigue, direct comparisons with SMW protocols were not performed and therefore such interpretations remain speculative. Another limitation is that the physiological mechanisms responsible for the observed PAPE effect remain speculative, as no direct measures of neural activation or muscle contractile function were obtained. On the other hand, inter-limb analyses were based on the classification of limbs as weaker or stronger based on CON. Although this approach allowed the source of asymmetry changes to be explored, it assumes stable between-limb dominance across conditions. Additionally, the SMH conditions provided participants with 1 and 5 additional minutes between the last warm-up set and the experimental repetitions compared to CON, and so the effect of SMH should be considered as a whole (including the SMH and recovery period), instead of in isolation. Also, we did not include within-session reliability measurements in the study design. Lastly, the Hawkin Dynamics system does not provide an automatic detection of the eccentric and concentric phases. Although segmentation was conducted in accordance with previous recommendations [38,39], the duration of the phases was extracted from the linear position transducer and a slight time lag may have existed in the T-Force system to detect the start and finish. However, since the return point was used to verify eccentric–concentric coupling and both the force-plates and T-Force sampled at the same high frequency (1000 Hz), we considered that the potential slight lag was consistent across repetitions with this approach rather than with a manual selection of eccentric start, return point, and concentric finish by the investigators. Using the return point as reference for the change from eccentric to concentric ensured that concentric RFD was not affected by phase segmentation.

Future research is warranted to assess the effect of SMH variations regarding load and rest before subsequent squats to further elucidate whether different balances of stimuli and fatigue affect the outcomes studied. Moreover, extending the analyses to stronger individuals such as high-level competitive powerlifters or weightlifters would provide further insights into the utility of the protocol. Nonetheless, addressing its effect against loads above 80% of 1RM would provide evidence of its utility in competitive contexts.

## 5. Conclusions

A single 10 s SMH coupled with a 1 min recovery period is a practical and costless strategy to enhance squat performance in strength-trained individuals. The enhancing effect is consistently reflected by improvements in concentric barbell velocity and force development, and is most evident after a 1 min recovery, while attenuated after a 5 min rest. In addition, postural stability remained largely unchanged across all conditions.

Therefore, practitioners seeking an acute enhancement in squat execution velocity may consider incorporating the 10 s SMH protocol at 110% of 1RM with a 1 min recovery period before subsequent squat sets.

## Figures and Tables

**Table 1:** Barbell kinematics during the concentric and eccentric phases of the back squat across loads in the control condition (CON) and following a 10 s supramaximal isometric hold at 110% of 1RM coupled with a 1 min (H1) or 5 min (H5) recovery.

Load (%1RM)	Condition (Group)	ROM (cm)	Con. MV (m·s^−1^)	Con. MPV (m·s^−1^)	Ecc. MV (m·s^−1^)	Ecc. PV (m·s^−1^)	Ecc. MP (W)	Ecc. PP (W)
60%	CON	65 ± 7	0.85 ± 0.04	0.88 ± 0.06	0.73 ± 0.22	1.27 ± 0.33	419 ± 139	838 ± 255
	H5	65 ± 8	0.88 ± 0.06	0.91 ± 0.06	0.70 ± 0.19	1.23 ± 0.29	402 ± 120	811 ± 232
	H1	65 ± 6	0.90 ± 0.07 *	0.93 ± 0.08 *	0.74 ± 0.22	1.25 ± 0.30	418 ± 110	829 ± 261
	Significance	*p* = 0.94	*p* = 0.01	*p* < 0.01	*p* = 0.74	*p* = 0.72	*p* = 0.87	*p* = 0.84
	ES (η^2^_p_)	<0.01	0.29	0.30	0.02	0.03	0.01	0.01
80%	CON	64 ± 5	0.64 ± 0.06	0.64 ± 0.06	0.58 ± 0.14	1.06 ± 0.23	437 ± 96	901 ± 258
	H5	64 ± 8	0.66 ± 0.05	0.66 ± 0.06	0.62 ± 0.15	1.04 ± 0.26	471 ± 111	914 ± 266
	H1	65 ± 7	0.68 ± 0.06 *	0.69 ± 0.06 *	0.66 ± 0.17	1.15 ± 0.26 *	497 ± 147	998 ± 291
	Significance	*p* = 0.82	*p* < 0.01	*p* < 0.01	*p* = 0.08	*p* = 0.02	*p* = 0.09	*p* = 0.06
	ES (η^2^_p_)	0.02	0.32	0.36	0.17	0.26	0.17	0.20

Con.: concentric; Ecc.: eccentric; ES: effect size; MP: mean power; MPV: mean propulsive velocity; MV: mean velocity; PV: peak velocity; PP: peak power; ROM: range of motion. * Significantly different (*p* < 0.05) from control condition in post hoc paired comparisons. Effect sizes are reported as eta partial squared (η^2^_p_).

## Data Availability

The dataset associated with this publication will be shared upon reasonable request to the corresponding author.

## Abbreviations

The following abbreviations are used in this manuscript:

1RM	One-repetition maximum
ANOVA	Analysis of variance
AP	Antero-posterior plane
CMJ	Counter-movement jump
CON	Control condition
COP	Center-of-pressures
ES	Effect Size
H1	Supramaximal isometric hold coupled with 1 min rest
H5	Supramaximal isometric hold coupled with 5 min rest
MF	Mean force
ML	Medio-lateral plane
MP	Mean power
MPV	Mean propulsive velocity
MV	Mean velocity
PAPE	Post-activation performance enhancement
PF	Peak force
PP	Peak power
PV	Peak velocity
RFD	Rate of force development
SD	Standard deviation
SMH	Supramaximal isometric hold
SMW	Supramaximal walkout
